# Influence of Surface Preparation of Aluminum Alloy AW-5754 and Stainless Steel X5CRNI18-10 on the Properties of Bonded Joints

**DOI:** 10.3390/ma17112561

**Published:** 2024-05-26

**Authors:** Nataša Zdravković, Damjan Klobčar, Dragan Milčić, Matevž Zupančič, Borut Žužek, Miodrag Milčić, Aleksija Đurić

**Affiliations:** 1 Faculty of Mechanical Engineering, University of Niš, Aleksandra Medvedeva 14, 18000 Niš, Serbia; natasa.zdravkovic@masfak.ni.ac.rs (N.Z.); dragan.milcic@masfak.ni.ac.rs (D.M.); miodrag.milcic@masfak.ni.ac.rs (M.M.); 2 Faculty of Mechanical Engineering, University of Ljubljana, Aškerčeva Cesta 6, 1000 Ljubljana, Slovenia; matevz.zupancic@fs.uni-lj.si; 3 Institute of Metals and Technology, Lepi Pot 11, 1000 Ljubljana, Slovenia; borut.zuzek@imt.si; 4 Faculty of Mechanical Engineering, University of East Sarajevo, Vuka Karadžića 30, 71123 East Sarajevo, Bosnia and Herzegovina; aleksija.djuric@ues.rs.ba

**Keywords:** adhesive bonding, surface preparation, aluminum alloy AW-5754, stainless steel X5CrNi18-10, epoxy adhesives

## Abstract

Adhesive bonding has proven to be a reliable method of joining materials, and the development of new adhesives has made it possible to use bonding in a variety of applications. This article addresses the challenges of bonding metals such as the aluminum alloy EN AW-5754 and the stainless steel X5CrNi18-10. In this study, the effects of laser cleaning and texturing on the surface properties and strength of two bonded joints were investigated and compared with mechanical preparation (hand sanding with Scotch-Brite and P180 sandpaper). The bonded joints were tested with three different epoxy adhesives. During the tests, the adhesion properties of the bonded surface were determined by measuring the contact angle and assessing the wettability, the surface roughness parameters for the different surface preparations, and the mechanical properties (tensile lap-shear strength). Based on the strength test results, it was found that bonded joints made of stainless steel had 16% to 40% higher strength than aluminum alloys when using the same adhesive and surface preparation. Laser cleaning resulted in maximum shear strength of the aluminum alloy bond, while the most suitable surface preparation for both materials was preparation with P180 sandpaper for all adhesives.

## 1. Introduction

Adhesives ensure a durable and stable connection by distributing the stress from one element to another much more evenly than mechanical fasteners. Adhesive bonding can often produce structures that are structurally comparable or even stronger than conventional joints, at lower cost and weight of the construction [1,2,3]. Compared to other, more traditional methods, adhesive bonding avoids stress concentrations and offers a high strength-to-weight ratio, excellent corrosion resistance, and ease of fabrication [4,5,6,7]. Many technical and mechanical factors [8,9] influence the quality and durability of bonded joints. These include the type of adhesive and the method of application, the shape and dimensions of a joint, and the type of load applied to a joint. The method of surface preparation prior to bonding is crucial for optimum joint performance and has a significant impact on the quality of the adhesively bonded joint. The surfaces of the parts to be joined (adherends) must be properly prepared. All contaminants must be removed from the surfaces to eliminate impurities or weak boundary layers (i.e., oxide layers), but also to improve the interaction between the adhesive and adherends [9,10,11]. The purpose of surface preparation is not only to remove contaminants but also to achieve maximum bond strength by increasing the difference in surface energy between the adhesive and the part to be bonded, resulting in good wetting and adsorption of the adhesive. Proper surface preparation must produce surface layers with sufficient strength to transfer loads throughout the life of the bonded joint.

A variety of mechanical, chemical, and other preparations have been developed to modify the surface properties of materials to achieve a cohesive failure mode and improved strength of bonded joints. Well-known surface preparations include mechanical abrasion [12,13], chemical etching [14,15,16], electrochemical methods—anodizing [17,18], plasma treatment [19,20], and laser treatment [21,22,23]. The surface preparation required for many adhesives goes far beyond simple cleaning or degreasing [24]. Although surface preparation can improve bond strength, degreasing alone is usually not sufficient to achieve good strength.

The surface preparation of metal bonding surfaces for bonding was discussed by Wasserbauer J, Pikner J [25], and Islam et al. [26]. It was found that bonding with proper surface preparation is a good bonding method due to the high strength of the bond under shear conditions. Safari et al. [27] investigated the influence of different surface preparations on the shear strength in the bonded joint. The results show that the strength of the lap joint increased steadily with increasing surface roughness of the sample. The conclusion is that precise measurement of surface roughness is an effective factor in controlling the quality of the design and manufacture of bonded joints. There are numerous scientific studies [28,29,30,31] on the effects of surface preparation on the strength of bonded joints using different adhesives and adherends. Among the papers published in the literature is the one by Łyczkowska et al. [32], in which the lowest adhesive bond properties were obtained with specimens degreased only with isopropyl alcohol before bonding, without any additional surface preparations. However, the indentations created by sanding with sandpaper increased the contact area of the adhesive and improved the properties of the joints. This indicates that the formation of larger indentations and the expansion of the surface layer improved both the shear strength and the fracture toughness of the bonded joint.

According to research by Dillard [33], aluminum alloys are traditionally considered “difficult to bond”. This is correct in that without adequate surface preparation of the aluminum surface prior to bonding, the strength would be poor, particularly with regard to the life of the joint. It is also claimed that various chemical and electrochemical methods increase the adhesion of stainless steel. According to Brockmann [34], etching methods are not recommended for the various types of steel, but mechanical removal methods such as hand sanding or sandblasting are generally used to achieve good adhesion results. Hand sanding is extremely inexpensive, requires less time and cost than other surface preparations, and does not involve some of the risks associated with compressed gas, electricity, or machinery [35]. While there are different types of sanding pads for hand sanding, the most commonly used ones in production are sandpaper with different grits and Scotch-Brite. It cuts and leaves smaller marks on the surface than sandpaper and generates less dust and dirt. The effect of surface preparation with Scotch-Brite has been sparsely studied in the past [36].

With the advances in laser technology, lasers have become increasingly important in surface preparation to improve surface roughness and thus the bond strength of adhesive joints. The laser method removes dirt and top coats without leaving any residue using only light. No chemicals or other media are required. The particles, which are produced in tiny quantities, can be removed and disposed of immediately after laser ablation. Many scientific studies have recently focused on understanding the behavior of adhesive bonds after the use of a laser on metallic materials. Rotella et al. [37] investigated the effect of laser ablation on steel adhesive bonds and showed that laser ablation not only improved the overall mechanical behavior of the adhesive joint under static loading but also maintained the strength of the bonded joint after exposure to aggressive environmental conditions. Laser ablation was used by Luo et al. and Rechner et al. to determine the strength of aluminum joints. Luo et al. [38] used laser ablation for texturing and creating suitable surface patterns, which significantly improved the bonding performance and increased the shear strength by 28.15% compared to the original samples. Rechner et al. [39] demonstrated improved strength and durability after laser ablation and showed how laser treatment improved surface cleaning while altering the oxide layer. Mandolfinoa et al. [40] investigated the laser cleaning treatment with different power ratios and pulse frequencies and achieved the highest values: up to double compared to the reference. The benefit of laser surface preparation is a combination of increased surface area, mechanical interlocking, and a change in surface chemistry. In fact, laser surface preparation helps to improve all these properties simultaneously. To the best of the authors’ knowledge, the influence of laser surface texturing on the strength of adhesive bonded joints and on the properties of aluminum alloy EN AW-5754 and stainless steel X5CrNi18-10 has not been completely investigated.

Based on the literature, the influence of surface preparation on joint strength has not been properly investigated, although it has a significant impact on the properties of bonded joints. A comparison of the maximum strength of joints of aluminum alloy EN AW-5754 and stainless steel X5CrNi18-10 prepared with P180 sandpaper and Scotch-Brite has rarely been considered, and no comparison has been made between different methods of surface preparation after laser cleaning and texturing. The aim of the present work was to investigate the influence of different surface preparations on the production of strong adhesive joints on two different adherends. The effects of surface roughness, topography, contact angle, failure mode on the experimental shear strength properties of four different surface preparations were investigated. This work helps to understand the bonding mechanisms of different surface preparations on different materials with different types of epoxy adhesives and provides a way to improve the bond strength of bonded joints.

## 2. Materials and Methods

### 2.1. Adherends

Aluminum alloy EN AW-5754 is a versatile metal alloy with a wide range of uses. It is known for its high strength-to-weight ratio, corrosion resistance, heat resistance, and good ductility and machinability—all properties that make it suitable for a wide range of applications in various industries. 

The second material studied, the austenitic chromium–nickel stainless steel X5CrNi18-10 (EN 1.4301), is characterized by high fracture and tear strength, low yield strength, and very high ductility and toughness values, and the element chromium gives it excellent corrosion resistance. Due to its properties of chemical and corrosion resistance, it is used in a wide range of instruments and devices, for industrial equipment, and as a construction alloy for the automotive, railroad, and aerospace industries, as well as a building material for large structures. The most important values of the chemical composition and mechanical properties of aluminum alloy and stainless steel are described in Table 1.

**Figure 1:**
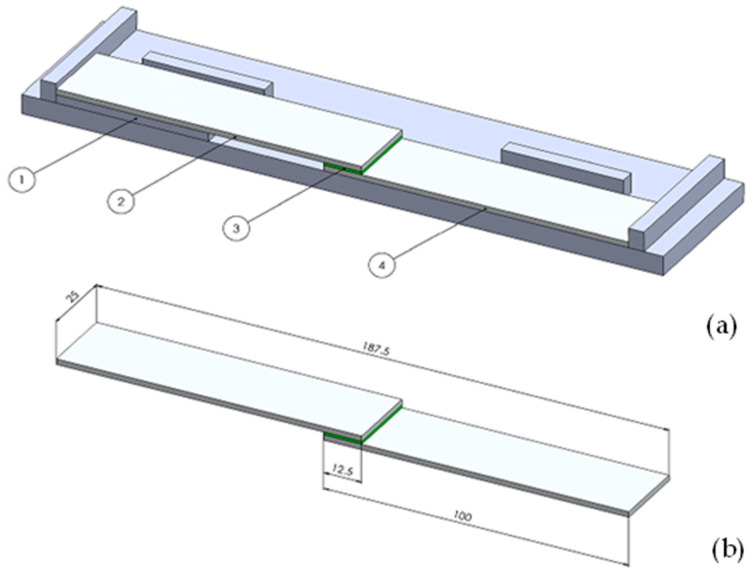
Mold for bonding tensile lap-shear joint (**a**) and dimensions of the test specimens for the single lap-shear test (**b**): (1) spacer, (2) upper test specimen, (3) adhesive layer, and (4) bottom test specimen.

The 2.0 mm thick sheets of cold-rolled aluminum alloy 5754 in the H22 state condition and stainless steel X5CrNi18-10 were prepared and cut into pieces (100 mm × 25 mm) with a waterjet according to the EN 1465 standard [41]. The specimens were used to produce joints that were overlapped (12.5 mm overlap) and adhesive bonded and then subjected to static shear tests. The alignment and holding mold was used to ensure correct positioning and overlapping of the specimens during the test. It provides up to 10 specimens, with the geometry shown in Figure 1a,b. The thickness of the adhesive during bonding was kept constant, with 0.3 mm aluminum plates as spacers (Figure 1a). The selected adhesives contained 0.3 mm-thick glass beads to additionally ensure optimum adhesive thickness.

### 2.2. Surface Preparation

During the tests, different preparations were used for the surface preparation of the parts to be joined prior to adhesive bonding, which were carried out on two different materials. Before mechanical processing, each specimen was carefully cleaned with SIKA Remover-208, a solvent-based cleaning agent for heavily soiled, non-porous surfaces, which is used to clean and degrease the surface before bonding. The surface preparation after cleaning is shown in Table 2.

The surfaces of the tested specimens were subjected to mechanical surface preparation, i.e., hand sanding. Sanding was carried out using fine-grained (green) Scotch-Brite sandpaper and P180-grit sandpaper. After sanding, all specimens were cleaned again with SIKA Remover-208 before bonding.

In addition, the surfaces were exposed to the effect of two lasers. The first laser was used to increase the surface energy of the material and clean the surfaces of the adherends, and the second to texturize the surface, i.e., to create different textures at different depths on the surfaces of the adherends.

Cleaning the surface with a laser before bonding is a non-contact, environmentally friendly cleaning method. As the energy concentration required to remove the impurities generated by the laser is much lower than the ablation threshold for metals, the surface of the base material remains unchanged. Laser ablation is the removal of a layer of a material or substance placed on a specified surface using a laser beam. Impurities can be removed selectively and with little impact on the adherends.

The Jetlaser M100 (Alsdorf, Germany) is a super compact and portable laser for cleaning small areas. The cleaning of specimens can begin without any prior preparation. The pulse energy was set to 1.25 mJ, the pulse overlapped to 8%, and the power to 45 W. The energy of the laser beam is very intense and extremely focused, which is why small shells or grooved structures can be created on the material in the processing zone. This modified surface is perfectly prepared for better mechanical adhesion. It also increases the surface tension of the material, which also contributes to better adhesive adhesion. Cleaning of the specimens can begin without prior preparation.

A nanosecond pulsed fiber laser was used for laser texturing (FL-mark-C with JPT Opto-electronics Co., Ltd., Warabi, Japan, “M7 30 W” MOPA source, *λ* = 1064 nm). The laser system is equipped with an OPEX F-Theta lens with a focal distance of 100 mm and a working area of 70 × 70 mm^2^. The process parameters that were kept constant during laser texturing were a pulse frequency of 37 kHz, a pulse width of 250 ns, and a power of 5%, with a focus beam spot diameter of ≈25 μm and a laser beam quality parameter M^2^ ≤ 1.3 (manufacturer data). The surface preparation patterns were designed in the “ezCAD 2” software, which was also used to control the laser system during laser preparation. 

**Figure 2:**
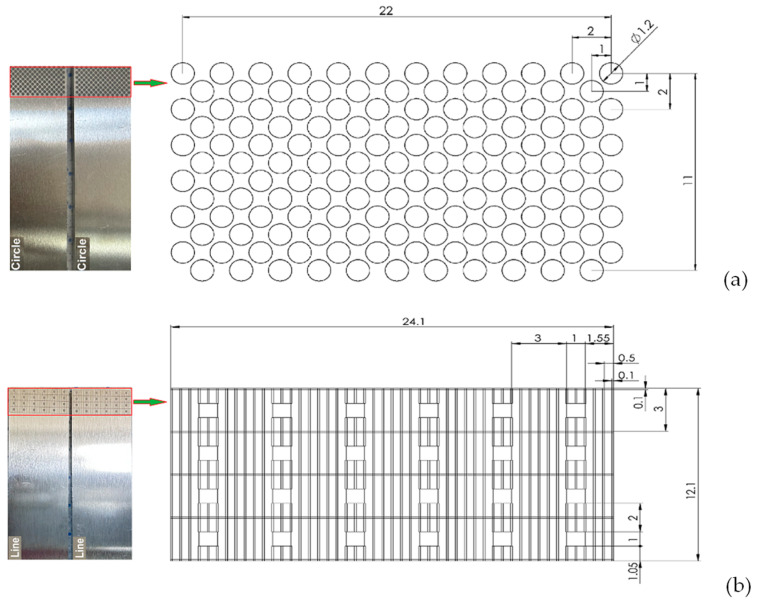
Laser textured patterns: (**a**) circle and (**b**) line.

To increase surface adhesion, two different patterns of textures were created at two different depths, 40 μm and 80 μm, in accordance with previous studies [42,43,44] on laser texturing and textures. The textures were created to introduce the effects that would affect bond strength. According to Maressa et al. [21], these effects include increasing the contact area, realizing mechanical interlocking by introducing micro-holes that can hold the adhesive, and realizing undercuts that can reduce the risk of adhesive failure by blocking the movement of the adhesive in the direction perpendicular to the surface of the material. The types of textures included two different patterns with the indicated effects, namely, circle (4C at a depth of 40 μm and 8C at a depth of 80 μm) and line (4L at a depth of 40 μm and 8L at a depth of 80 μm) (Figure 2).

The first structured pattern, circle, consisted of ordinary micro-holes (Figure 2a). The primary function of these micro-holes would be to improve the surface and mechanical interlocking perpendicular to the shear direction. Laser surface texturing in this form is achieved by a punctual percussive drilling process. Texturing of circular surfaces can be extremely productive with the correct choice of parameters and the use of a limited number of pulses [21]. The hole diameter is proportional to the size of the laser beam and, together with the hole depth, is controlled by the laser power and the number of pulses or the number of consecutive passes. The number of consecutive passes is different for each material and texture and was determined after the corresponding depths of 40 μm and 80 μm was reached. Table 3 shows the number of consecutive passes for both materials and textures. Line patterns, the second texture, were intended to represent overlapping pits [42]. The line pattern was created with the aim of achieving a surface pattern with a larger surface area for touch and mechanical interlocking (Figure 2b). The line texture was created by digging perpendicular lines on the material surface.

### 2.3. Adhesives

Three types of adhesives were selected for this study, namely, the two-component (2C) epoxy adhesive SikaPower^®^-880, a high fatigue-resistant adhesive intended for structural joints where toughness and high strength are required. The epoxy adhesive was applied to the cleaned surfaces and then cured in a drying and heating chamber [45]. The drying and heating chambers were manufactured by ED 56 Binder GmbH. The second of the adhesives selected for the tests is a so-called epoxy hybrid adhesive SikaPower^®^-492 G, a one-component (1C), high-strength, and crash-resistant structural adhesive suitable for highly structural bonding of various types of metal. The epoxy-based adhesive was heated to a temperature of 55 °C for 15 min, then applied to the surfaces and cured in an oven [46]. Finally, SikaFast^®^-580 is a high temperature acrylic/epoxy hybrid [47]. It is characterized by high-strength development within minutes after application. The adhesive should be applied to appropriate surfaces within 4 to 7 min (“open time”). All three adhesives are general purpose and are used for bonding metals. Selected mechanical properties of the adhesives are described in Table 4. After the curing time, the joints were removed from the chamber. A digital caliper (Mitutoyo 500-196-30) was used to check the dimensions and the strength of the bonded joints was tested.

### 2.4. Microscopic Analysis of the Surface Topography Prepared for Bonding

A Thermo Scientific Quattro ESEM (Tokyo, Japan) scanning electron microscope (SEM) was used to analyze the microstructure of the surfaces created by the different types of bonding preparation. The tests were performed at magnifications of up to 1000 using the secondary electron and backscattered electron techniques. Specimens of 20 mm × 25 mm × 2 mm were prepared for chemical composition analysis. Before analysis, all specimens were washed in an ultrasonic bath with ethanol.

A Keyence VHX-6000 (Keyence Corporation, Itasca, IL, USA) digital microscope was used for engraving analysis and measurement of laser parameters. The microscope can analyze the surface topography and image materials at magnifications of 20× to 2000×.

The surface roughness of the specimens prepared with Scotch-Brite and P180 sandpaper was measured using the contact method and a profilometer (Mitutoyo, Sakado, Japan, type SJ-301) with a two-dimensional surface registration technique.

### 2.5. Contact Angle Measurement

The wettability of the surfaces was evaluated by measuring the static contact angle using double distilled water at room temperature to investigate its influence on the adhesion of two adherends. The Osilla Contact Angle Goniometer (Osilla, Sheffield, UK) was used for the contact angle measurements. 

### 2.6. Strength Test

**Figure 3:**
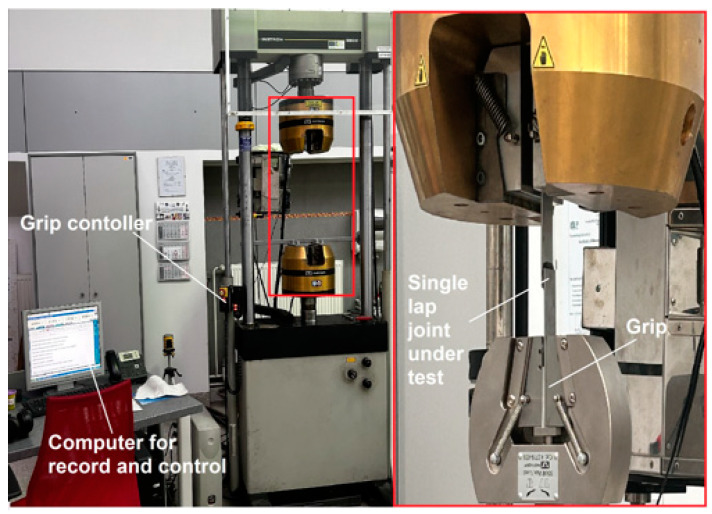
Setup for the shear test with a single lap joint on Instron 8802.

The single-lap adhesively bonded joints were tested under shear conditions. The adhesion was determined by the tensile lap-shear test according to the ISO 4587 standard [48]. The lap shear tests of the lap joints were performed at room temperature using an INSTRON 8802 250 kN (Norwood, MA, USA) testing machine under displacement control at a test speed of 1 mm/min. At least three specimens per joint type were tested until complete failure. The nominal lap shear strength (in MPa) was calculated by the ratio between the maximum force and the adhesively bonded area of the joints. The dimensions of the joints were measured on fully cured joints. The setup for the single lap-shear test is shown in Figure 3.

### 2.7. Analysis of the Fractured Bonding Surfaces

After the tension lap-shear test, the fracture surfaces were analyzed. The specimens were photographed with a high-resolution digital camera and processed with ImageJ image processing software (1.8.0., National Institute of Health, Bethesda, MD, USA).

## 3. Results and Discussion

### 3.1. Analysis of Surface Topography Prepared for Bonding

**Figure 4:**
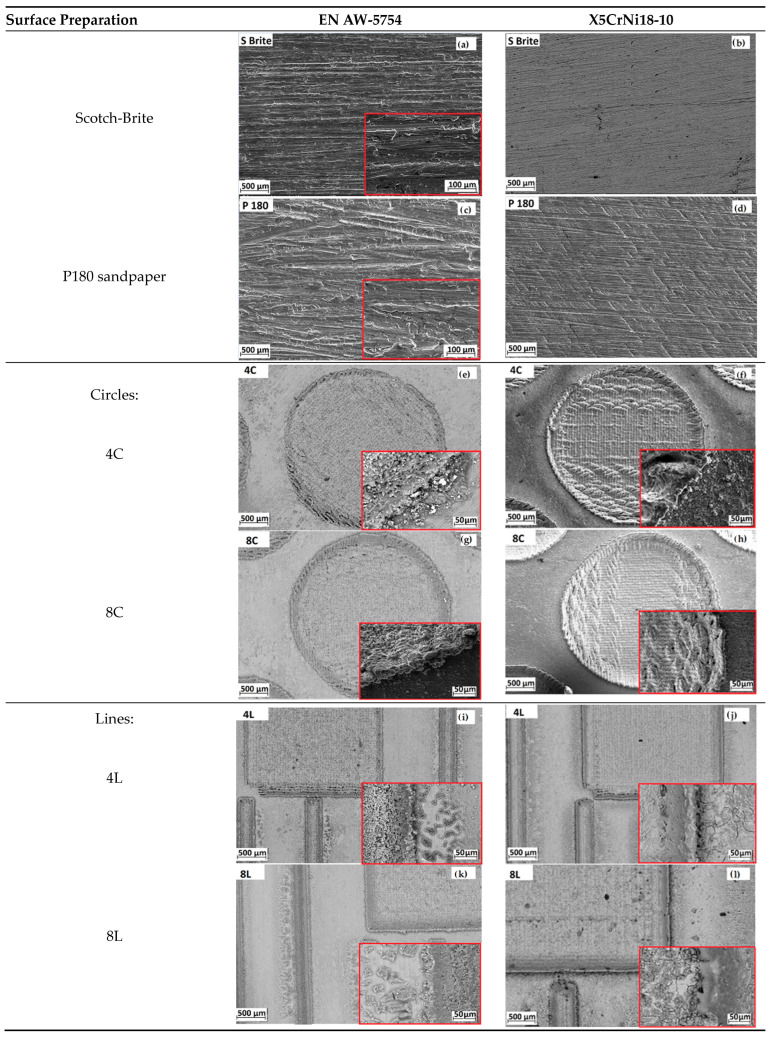
SEM images of surfaces: (**a**,**b**) hand sanding with Scotch-Brite; (**c**,**d**) hand sanding with P180 sandpaper; (**e**,**f**) laser texture circle with a depth of 40 µm; (**g**,**h**) laser texture circle with a depth of 80 µm; (**i**,**j**) laser texture line with a depth of 40 µm; (**k**,**l**) laser texture line with a depth of 80 µm. Scale bars in the inset images: (**a**,**c**) 100 µm; (**e**–**l**) 50 µm.

Figure 4 shows SEM and focus variation microscopy photographs of hand sanding and two laser-textured surfaces at two depths. A larger area of the surfaces prepared with Scotch-Brite and with P180 sandpaper are shown due to the greater surface roughness. The P180 sandpaper, with its coarser grit, leads to deeper scratches and a higher surface roughness (Figure 4c). In contrast, intensive heating and a melting, spraying, and freezing process of the material can be observed on the surfaces of the specimens that were exposed to the laser pulses. This resulted in the formation of a processed layer on the surface of the untreated material in the form of bumps around the textures, as highlighted by Yang et al. [49]. This indicates a deviation from the design, which can have both positive and negative effects.

The first textured pattern, a circle, was characterized by micro-holes of 40 μm and 80 μm depth. The amount of molten material was squeezed out of the hole and interacted with the surrounding area or with other streams of molten material ejected nearby. As it cooled and solidified, it fused into a solid compound that bonded to the surface near the laser interaction. Finally, a spike formed, which was concentrated around the entrance of the hole, between and within the micro-holes, and increased with increasing texture depth (Figure 4e–h). This can help with mechanical interlocking, but it can also lead to changes in wetting and increase the overall surface roughness. However, as reported by Maressa et al. [21], the adhesion of the recast material layer to the bulk material may be weaker than the adhesion to the adhesive.

The line texture showed well-defined indentations on the surface and deviations from the desired shapes due to the processed layer on the material surface. The line pattern had the greatest influence on the surface morphology, as its structure was more complicated and the proportion of the laser-processed area was also larger, as can be seen in Figure 4i,k for aluminum alloy and Figure 4j,l for stainless steel. The distance between the lines was 0.5 mm and each line was 0.1 mm wide, which had a considerable influence on the surface topography. With increasing depth, a high number of bumps on the aluminum alloy (Figure 4k) spikes and bubbles on the stainless steel (Figure 4l), appeared around the surface line texture, indicating the fractal structure of the processed material, which can cause differences in surface wetting. Laser processing, on the other hand, resulted in no mechanical deformation of the material and had the least processing error. Under the microscope, there was no significant difference between the holes or lines at the end of the overlapping area and those in the middle of both materials.

In addition, the effects of these different types of microstructures on the chemical composition, surface roughness, and surface contact angle differed, as will be explained in the following sections. 

Different surface preparations can influence the chemical composition of the surface. Table 5 and Table 6 show the chemical composition of the surface after different surface preparations. After preparation with P180 sandpaper and Scotch-Brite, the percentage of the elements C and O did not change significantly and the O content was lower in both materials, especially the stainless steel. The content of Al (for EN AW-5754) and Fe (for X5CrNi18-10) after laser texturing decreased and the content of element O increased compared to the other preparations, indicating that the aluminum alloy EN AW-5754 and the stainless steel X5CrNi18-10 were oxidized and generated on the surface during laser texturing. Changes in the chemical composition of the surface subsequently affect the change in strength and surface wettability over time.

### 3.2. Analysis of Surface Roughness

**Figure 5:**
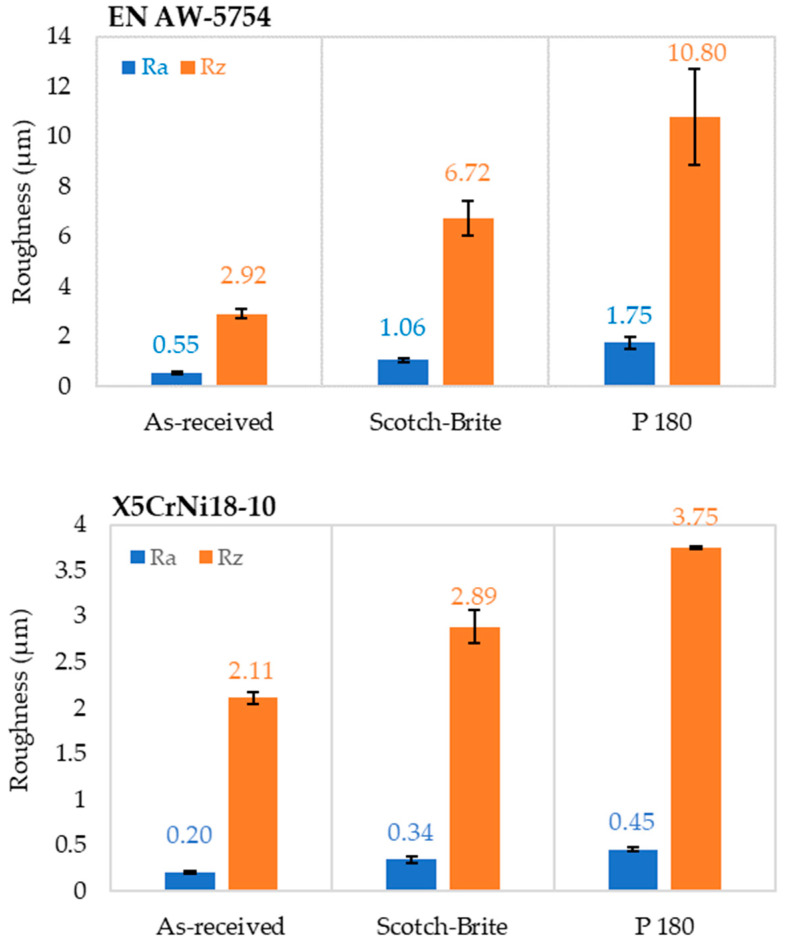
Average surface roughness of the aluminum alloy EN AW-5754 and the stainless steel X5CrNi18-10 depending on the surface preparation.

The surface roughness of the specimens was evaluated using two roughness parameters: average roughness (Ra) and maximum roughness (maximum profile height, Rz). Measurements were taken at several points in two mutually perpendicular directions, longitudinally and tangentially. The measurements were carried out in three separate measurement regions for each specimen. Two depths of 40 µm and 80 µm were defined for the laser-prepared specimens. Figure 5 shows the measurement results for the two primary surface roughness parameters Ra and Rz in relation to the individual types of surface preparation.

In the case of aluminum alloys, a significant increase in bond strength was achieved for the specimens prepared with P180 sandpaper compared to the specimens prepared with Scotch Brite. The average Ra and Rz values were ±1.75 µm (1.46–1.91 µm) and ±10.8 µm (8.65–12.33 µm), respectively. For X5CrNi18-10, the average Ra and Rz values were ±0.45 µm (0.43–0.48 µm) and ±3.75 µm (3.74–3.76 µm), respectively. As a result, the surfaces of both materials prepared with P180 sandpaper exhibited a higher surface roughness.

### 3.3. Wettability

**Figure 6:**
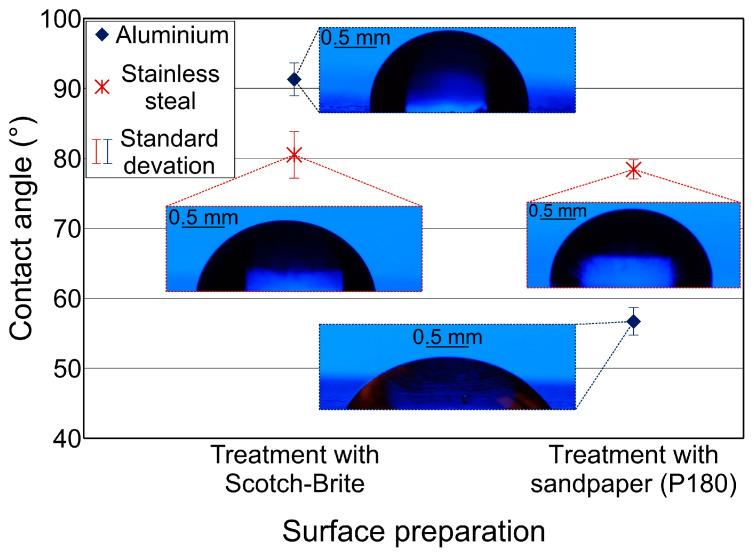
Average contact angle values for the aluminum alloy EN AW-5754 and the stainless steel X5CrNi18-10 depending on the surface preparation.

Figure 6 shows the average contact angle values of the different surface preparations, the surface prepared with Scotch-Brite and the surface prepared with P180 sandpaper. The surfaces prepared with Scotch-Brite showed higher contact angles compared to the surfaces prepared with P180 abrasive paper, indicating a near-neutral wetting condition. This discrepancy can be attributed to the different degree of surface roughness exhibited by the two preparations. In particular, sandpaper P180 with its coarser grit led to deeper scratches and a higher surface roughness. Consequently, the greater roughness increased the total surface energy, which facilitated the wetting of the liquid droplets and led to a lower contact angle.

**Figure 7:**
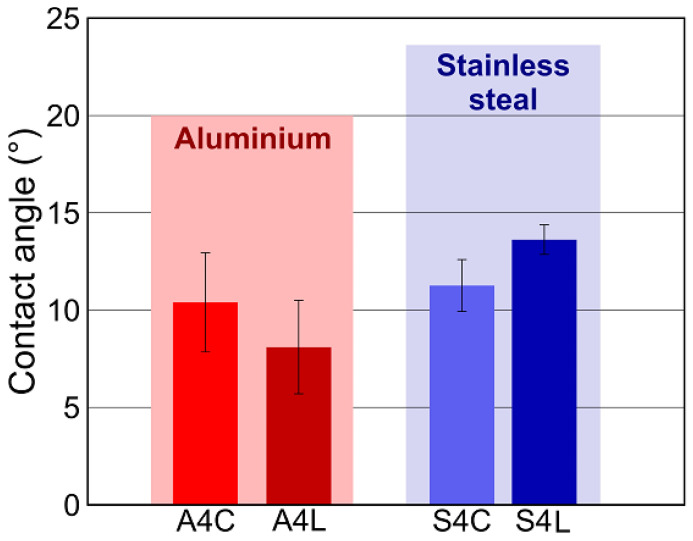
The contact angles after time intervals for the aluminum alloy surfaces after 12 days and for the stainless steel surfaces after 3 days.

Furthermore, both aluminum alloys and stainless steel surfaces exhibited a superhydrophilic wetting state after the laser texturing process, which was characterized by contact angles close to 0°. In particular, two different depths of laser texturing, namely, 40 μm and 80 μm, were used. To simulate real industrial applications and evaluate the behavior of these laser-textured surfaces in terms of adhesion, the 40 μm laser-textured surfaces were exposed to ambient air for different periods of time. In particular, the stainless steel surfaces were exposed for 3 days, while the aluminum surfaces were exposed for 12 days. The contact angles measured on these surfaces after the specified time intervals are shown in Figure 7.

The results indicate a gradual increase in contact angle over time, suggesting a transition from the initial superhydrophilic state to a hydrophilic state after several days in ambient air. This change in wettability has been observed in numerous previous studies and can be attributed to the adsorption of hydrophobic contaminants in the atmosphere. Recent studies have emphasized the importance of adsorption of polymeric organosilicon compounds as the most likely explanation for the change in wettability of such samples in both laboratory and industrial environments [50,51].

In addition, Figure 7 shows a notable disparity in the rate of contact angle increase over time between aluminum alloy and stainless steel surfaces. For instance, considering surfaces A4L and S4L, the contact angle on surface S4L reached 13.6° after a three-day exposure to ambient air, while specimen A4L exhibited a contact angle of 8.1° following a 12-day exposure period. This discrepancy in the change in contact angle, with stainless steel surfaces demonstrating a more pronounced alteration in a shorter time of exposure to ambient air compared to aluminum surfaces, can be attributed to the distinct oxidation tendencies and varying free energy of these two materials. Consequently, it led to differences in the adsorption behavior of polymeric organosilicon compounds, thereby resulting in variations in the observed contact angles.

### 3.4. Tensile Lap-Shear Strength

**Figure 8:**
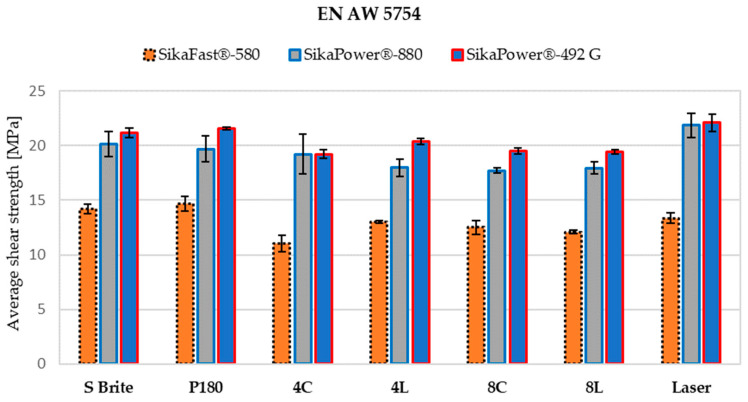

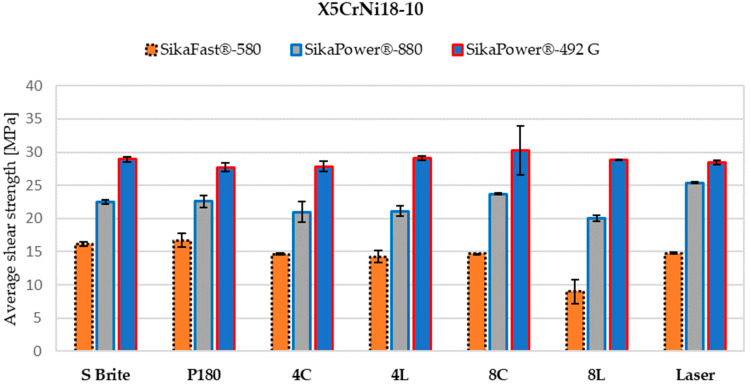
Average shear strength of adhesive-bonded joints in relation to the method of surface preparation for aluminum alloy and stainless steel. The first bar shows bonded parts prepared with Scotch-Brite; the second bar shows bonded parts prepared with P180 sandpaper; the third bar shows a laser texture circle with a depth of 40 μm; the fourth bar shows a laser texture line with a depth of 40 μm; the fifth bar shows a laser texture circle with a depth of 80 μm; the sixth bar shows a laser texture line with a depth of 80 μm; the seventh bar shows a laser cleaning preparation.

The results of the static tensile shear test of the single lap-bonded joints in relation to different materials, surface preparations, and adhesive types are shown in the form of a graph in Figure 8. The results show that surface preparation with Scotch-Brite and P180 sandpaper had an influence on the generation of sufficient surface roughness, as did the effect of the laser on increasing the surface energy. The analysis of the results of the static shear tests show that there were specific dependencies that were independent of the materials and adhesives used. There were correlations between the preparation, the adhesives, and the strength of the bonded joint. 

For 2C adhesives, the highest strengths were observed for both materials when the surfaces were prepared with the same preparation method, for adhesive 580 with P180 sandpaper and for adhesive 880 with laser cleaning. 

Adhesive 580 had a lower strength than other adhesives, although the samples prepared with sandpaper P180 had a higher strength than the samples prepared by laser texturing or even cleaning. Laser cleaning increased the mechanical strength of bonded joints with adhesive 880 by 2 to 5 MPa compared to the values obtained with alternative preparations.

The maximum strength of one-component adhesive 492 varied depending on the material and preparation method. Laser cleaning on aluminum increased the strength by up to 3 MPa compared to the weakest results obtained with laser texturing. For stainless steel, the strength after laser cleaning was in the range of the strength achieved with P180 sandpaper, while laser texturing proved to be the best method of surface preparation for this adhesive and material.

The results show that the 880 adhesive was significantly more sensitive to the method of surface preparation, with a considerable deviation in bond strength of up to 5 MPa. By cleaning the surface appropriately, the method described above resulted in suitable surface expansion and therefore increased surface adhesion.

Significant differences in strength were found between joints of aluminum alloy EN AW-5754 and stainless steel X5CrNi18-10 when the same adhesive and surface preparation were used. Stainless steel joints with the same epoxy adhesives exhibited 16% to 40% higher strength than aluminum alloy joints, depending on the choice of surface preparation. The differences in strength between materials were mainly due to the different properties of the individual metal alloys surfaces, which resulted in different degrees of adhesion to each part to be bonded. Selecting a surface preparation that is suitable for the type of parts to be joined and their surface roughness is of great importance, as it can improve the strength of the bonded joints. 

### 3.5. Failures Mode Surface

Appropriate surface preparation should ensure that the least strong bond in an adhesive joint is within the adhesive layer and not at the contact of the bonding surfaces. This investigation has helped shed light on the causes of failure and draw some conclusions about the quality of bonded joints. The failure mode according to ISO 10365 [52] is used to classify failures in order to better understand the result of a mechanical adhesion test on an adhesive bond, which is generally expressed in quantitatively measurable values. Table 7 provides details of the failure mode designations. In this study, the failure mode for all surfaces was observed immediately after the static shear test. 

**Figure 9:**
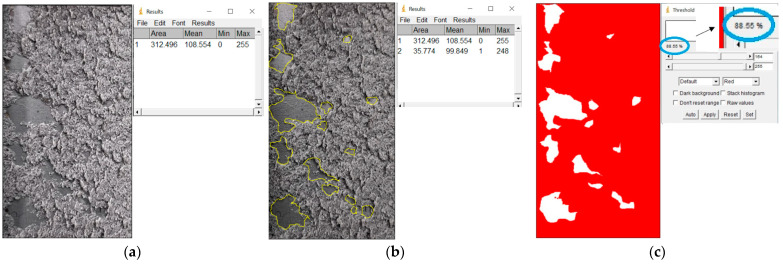
The method for deriving fracture mode percentages. (**a**) Photograph of the examined fracture surface; (**b**) processing of the photo with ImageJ software; (**c**) color scale-based determination of the failure mode percentage.

The fracture mode of adhesive bonds differed depending on the materials used, the surface preparations, and the adhesives used. The percentage of area was measured using ImageJ software. The total measured bond area was 312.496 mm^2^ (Figure 9a), and the area of the fracture surface without adhesive was 35.774 mm^2^ (Figure 9b). The percentage of area in mode of failure was calculated by dividing the area of adhesion failure by the total bonded area and multiplying by 100. The areas of the fracture surface that had a layer of adhesive on the fracture surface are colored red (Figure 9c), and they accounted for 88.55% of the CF, whereas the areas without adhesive accounted for 11.45% of the AF. Based on this simple procedure, the percentages of the failure modes were derived and are shown for all samples.

**Figure 10:**
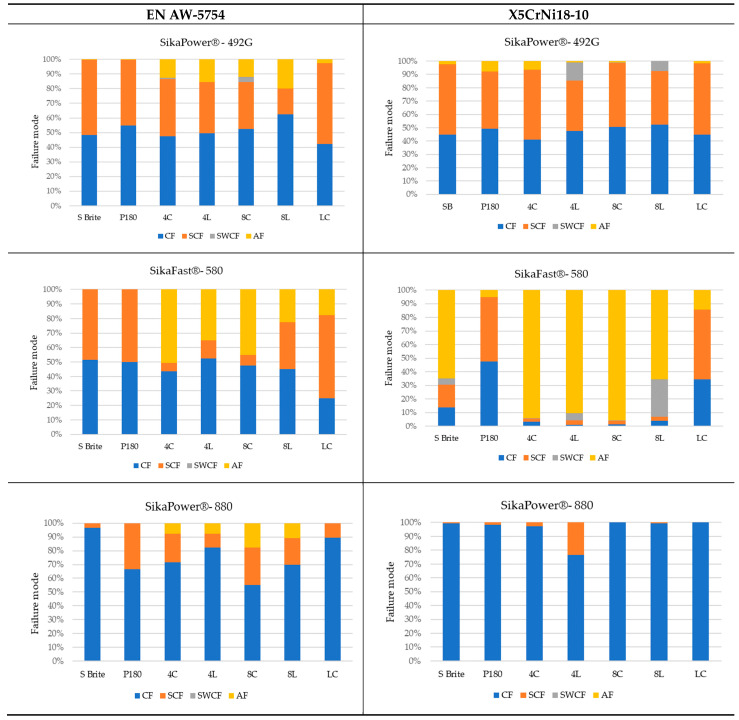
Percentage of failure mode distribution of the SikaPower^®^-492 G, SikaFast^®^-580, and SikaPower^®^-880 adhesives for the aluminum alloy EN AW-5754 and the stainless steel X5CrNi18-10 depending on the surface preparation. CF—cohesion failure; SCF—special cohesion failure; SWCF—failure with stress whitening of adhesive; AF—adhesion failure.

Figure 10 shows examples of fractures in bonded joints made with the adhesives SikaPower^®^-492 G, SikaFast^®^-580, and SikaPower^®^-880. Cohesive failure or special cohesive failure dominated with the 1C adhesive 492 for both materials. However, adhesion failure was observed in the aluminum alloy after laser texturing and in the stainless steel after the application of P180 sandpaper, which is certainly related to the fact that the results of the static shear test showed low strength in these surface preparations with this adhesive. For the 2C 580 adhesive, adhesive failure dominated after surface preparation by laser texturing for both materials, leading to lower strength results, in contrast to the preparation with Scotch-Brite and P180 sandpaper, where cohesive failure dominated and increased the shear strength value. With the exception of the aluminum alloy, where adhesive failure occurred after laser texturing, the 2C adhesive 880 gave very consistent results, mostly cohesive failure. The fact that this adhesive reacted very sensitively to different surface preparations and that the strength determined after the test was different proves that this adhesive leads to adhesive failure with both materials in all preparations.

With regard to the preparation, it can be deduced for both materials that the fracture of the adhesive was more pronounced in specimens that were prepared with P180 sandpaper, Scotch-Brite, or laser cleaning. In most cases, adhesion failure occurred after laser texturing due to insufficient adhesion, contamination, or splattering after laser texturing, indicating that the aluminum alloy EN AW-5754 and stainless steel X5CrNi18-10 were oxidized and generated on the surface during laser texturing. 

## 4. Conclusions

In this study, four different surface preparations were applied to the aluminum alloy EN AW-5754 and the stainless steel X5CrNi18-10 to determine the influence of the different surface preparations on the properties of bonded joints with three different epoxy adhesives. The following conclusions were drawn based on the investigations carried out and the examination of the knowledge gained:

- The removal of surface impurities and the uniform development of the bonding surface were associated with a higher strength of the bonded joint as well as good wetting and adsorption of the adhesive.
- Laser cleaning as a surface preparation for bonding resulted in maximum shear strength of the bonded joint (an increase over hand sanding and laser texturing), while the most suitable surface preparation for aluminum alloy and stainless steel joints was preparation with P180 sandpaper for all adhesives. Slightly better results were achieved with laser texturing for stainless steel joints for SikaPower^®^-492 G adhesive.
- The application of SikaPower^®^-880 2C epoxy adhesive for bonding materials showed optimal results for all preparations and materials, with results showing that adhesive 880 was significantly more sensitive to the surface preparation method, having the highest shear strength and the highest percentage of cohesive failure for both materials.
- Using the same surface preparation methods and adhesives, X5CrNi18-10 stainless steel-bonded joints had 16% to 40% higher strength than EN AW-5754 aluminum alloy joints, indicating that choosing the right surface preparation methods with appropriate surface roughness can improve the strength of bonded joints.

## Figures and Tables

**Table 1:** Main chemical constituents and mechanical properties of aluminum alloy EN AW-5754 and stainless steel X5CrNi18-10.

Materials	Chemical Components (%)									
EN AW-5754	Si	Fe	Cu	Mn	Mg	Cr	Zn	Ti	other	Al
	0.26	0.26	0.04	0.25	2.81	0.04	0.03	0.01	0.15	Balance
X5CrNi18-10	C	Cr	Ni	Mn	Si	Cu	N	P	S	Fe
	0.026	18.17	8.05	1.28	0.34	0.27	0.063	0.024	0.001	Balance
**Materials**	**Mechanical Properties**									
	**Yield Strength (MPa)**			**Tensile Strength (MPa)**		**Elongation to Break (%)**			**Module of Elasticity (GPa)**	
EN AW-5754	180			236		16.5			70	
X5CrNi18-10	316			657		57.1			200	

**Table 2:** Surface preparation of aluminum alloy EN AW-5754 and stainless steel X5CrNi18-10.

Surface Preparation	Details
Scotch-Brite (hand sanding)	Sand the bonded surface evenly by hand with green fine-grit Scotch-Brite sandpaper for about 30 s in one axial direction with medium pressure, and then apply SIKA Remover-208 to clean the bonded surface before bonding.
P 180 (hand sanding)	Sand the bonded surface evenly by hand with 180-grit sandpaper for about 30 s in one axial direction with medium pressure, and then apply SIKA Remover-208 to clean the bonded surface before bonding.
LC (laser for cleaning)	Use a laser surface preparation machine with a power setting of 45 W and a pulse energy of 1.25 mJ to clean the bonding surface thoroughly.
LT (laser for texturing)	The untreated bonded surface is treated with a nanosecond pulsed fiber laser for laser texturing with a constant power of 5% and a limited number of pulses for each texture and depth (40 μm and 80 μm).

**Table 3:** Number of consecutive passes for laser-textured forms.

Material	Laser Textured Forms	40 μm	80 μm
EN AW-5754	Circle	7	15
	Line	14	38
X5CrNi18-10	Circle	24	45
	Line	42	110

**Table 4:** Mechanical properties of the adhesives [45,46,47].

Adhesive	SikaPower^®^-880	SikaPower^®^-492G	SikaFast^®^-580
Viscosity (Pa·s)	100	230	150
Tensile strength (MPa)	22	29	12
Elongation at break (%)	3.3	8	15
Module of elasticity (MPa)	2220	2190	900

**Table 5:** Chemical composition measured using a scanning electron microscope EDS analysis of the aluminum alloy EN AW-5754 depending on the surface preparation (Weight %).

Elements	Al	O	C	Mg	Fe
Scotch-Brite	91.12	3.82	1.27	2.81	0.98
P 180	92.44	2.31	1.81	2.83	0.61
4C	83.84	8.69	2.1	3.58	1.79
4L	85.02	7.02	3.37	3.39	1.2
8C	73.03	17.89	1.6	3.79	3.69
8L	72.28	18.74	1.46	3.63	3.89

**Table 6:** Chemical composition measured using a scanning electron microscope EDS analysis of the stainless steel X5CrNi18-10 depending on the surface preparation (Weight %).

Elements	Fe	O	C	Cr	Ni
Scotch-Brite	70.45	1.09	2.2	18.53	7.73
P 180	71.43	0.42	1.54	18.39	8.22
4C	64.95	8.47	2.2	18.39	5.99
4L	63.08	10.5	2.35	17.47	6.6
8C	64.41	8.83	1.98	17.52	7.26
8L	57.82	18.31	2.73	15.39	5.75

**Table 7:** Examples of cohesive and adhesive failures [52].

**Cohesion Failure (CF)**	**Failure with Stress Whitening of Adhesive (SWCF)**
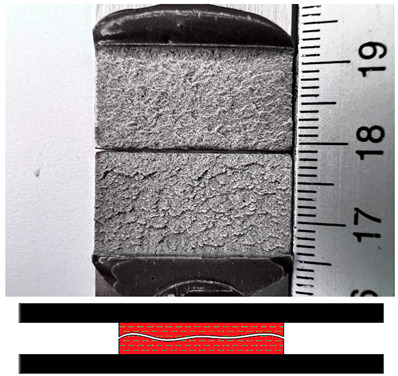	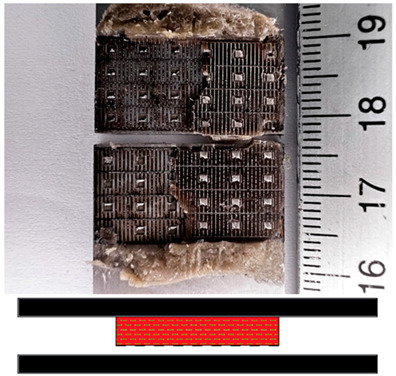
**Special Cohesion Failure (SCF)**	**Adhesion Failure (AF)**
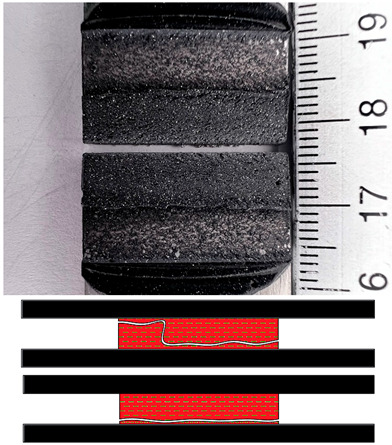	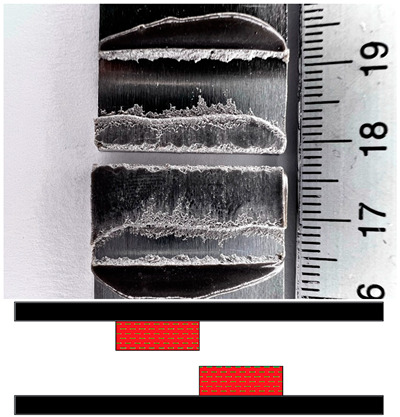

## Data Availability

The data supporting reported results are not stored in any publicly archived datasets. The readers can contact the corresponding author for any further clarification of the results obtained.
